# Educational interventions alone and combined with port protector reduce the rate of central venous catheter infection and colonization in respiratory semi-intensive care unit

**DOI:** 10.1186/s12879-019-3848-z

**Published:** 2019-03-04

**Authors:** Riccardo Inchingolo, Giuliana Pasciuto, Daniele Magnini, Manuela Cavalletti, Giancarlo Scoppettuolo, Giuliano Montemurro, Andrea Smargiassi, Riccardo Torelli, Maurizio Sanguinetti, Teresa Spanu, Giuseppe Maria Corbo, Luca Richeldi

**Affiliations:** 1 UOC Pneumologia, Fondazione Policlinico Universitario A. Gemelli IRCCS, Rome, Italy; 2 UOC Malattie Infettive, Fondazione Polcilinico Universitario A. Gemelli IRCCS, Rome, Italy; 3 UOC Microbiologia, Fondazione Policlinico Universitario A. Gemelli IRCCS, Rome, Italy; 4 Istituto di Microbiologia, Università Cattolica del Sacro Cuore, Rome, Italy; 5 UOC Pneumologia, Università Cattolica del Sacro Cuore, Rome, Italy

**Keywords:** Venous central catheter, Central line-associated bloodstream infections, Port protector, Respiratory semi-intensive care unit

## Abstract

**Background:**

Central Line-Associated BloodStream Infections (CLABSIs) are emerging challenge in Respiratory semi-Intensive Care Units (RICUs). We evaluated efficacy of educational interventions on rate of CLABSIs and effects of port protector as adjuvant tool.

**Methods:**

Study lasted 18 months (9 months of observation and 9 of intervention). We enrolled patients with central venous catheter (CVC): 1) placed during hospitalization in RICU; 2) already placed without signs of systemic inflammatory response syndrome (SIRS) within 48 h after the admission; 3) already placed without evidence of microbiologic contamination of blood cultures.

During interventional period we randomized patients into two groups: 1) educational intervention (Group 1) and 2) educational intervention plus port protector (Group 2).

We focused on CVC-related sepsis as primary outcome. Secondary outcomes were the rate of CVC colonization and CVC contamination.

**Results:**

Eighty seven CVCs were included during observational period. CLABSIs rate was 8.4/1000 [10 sepsis (9 CLABSIs)]. We observed 17 CVC colonizations and 6 contaminations. Forty six CVCs were included during interventional period. CLABSIs rate was 1.4/1000. 21/46 CVCs were included into Group 2, in which no CLABSIs or contaminations were reported, while 2 CVC colonizations were found.

**Conclusions:**

Our study clearly shows that both kinds of interventions significantly reduce the rate of CLABSIs. In particular, the use of port protector combined to educational interventions gave zero CLABSIs rate.

**Trial registration:**

NCT03486093 [ClinicalTrials.gov Identifier], retrospectively registered.

## Background

Central Line-Associated BloodStream Infections (CLABSIs) are responsible for as many as 28,000 deaths in the United States annually [1–7]. A recent analysis found that 65–70% of CLABSIs could be prevented with the proper institution of catheter care measures [8].

There are 2 main routes by which central venous catheters (CVCs) become colonized: from bacteria contaminating the outside of the catheter either at the time of insertion or after insertion (extraluminal) and/or by contamination of the hub, catheter, or other administration device (intraluminal). The extraluminal pathway predominates in the first week after CVC placement, whereas the intraluminal mechanism is the more common pathway for CVCs that are > 1 week old [9, 10].

Several collaboratives have demonstrated the preventability of these infections [11, 12]. Educational interventions decrease rates of CLABSIs [13]. In addition to training, education, and surveillance, important prevention practices include the use of chlorhexidine skin antiseptics and maximal sterile barrier precautions at catheter insertion [14, 15].

Other maintenance practices include hand hygiene before handling catheters or catheter sites, chlorhexidine for skin antisepsis with dressing changes, and disinfecting catheter hubs or injection ports with an appropriate agent before accessing the catheter [16, 17].

Non antibiotic antiseptic locks (such as alcohol or trisodium citrate), have also demonstrated some success in reducing CLABSIs. In particular, alcohol-impregnated port protectors and needleless neutral pressure connectors significantly reduced rates of CLABSIs [18].

To date, in literature, no data exist on rate, risk factors and prevention strategies of CLABSIs in RICUs. These units usually work as “step-up” units within acute care hospitals to manage patients with respiratory failure with non-invasive ventilation [19, 20]. These units may provide multidisciplinary rehabilitation [21] and serve as a bridge to home-care programs or long-term care facilities [22]. Some of these RICUs may work also as “step down” units for difficult to wean patients [23].

We performed a single-centre prospective study with the aim to assess the efficacy of educational interventions alone and combined with port protector as adjuvant tool on rate of CLABSIs (primary outcome). Secondary outcomes were the effects of previously mentioned interventions on rates of CVC colonizations and contaminated blood cultures.

## Methods

This study was approved by the Local Ethical Committee of the Catholic University of the Sacred Heart in Rome, Italy (Prot.n°5664) and it was performed according to the Helsinki Declaration of Good Clinical Practice. Three departments were involved: Respiratory semi-Intensive Care Unit, Institute of Microbiology and Institute of Infectious Diseases.

### Participants

The study enrolled patients admitted to RICU from April 2013 and it lasted 18 months. We chose three mutually exclusive inclusion criteria were: 1) CVC was placed during hospitalization in RICU; 2) CVC already in place at admittance without signs of systemic inflammatory response syndrome (SIRS) during first 48 h from admission to RICU [24]; 3) CVC already placed at admittance without evidence of microbiologic contamination of blood cultures.

Each patient gave written informed consent.

Patients enrolled underwent blood cultures sampling whenever they showed SIRS signs.

SIRS is defined as 2 or more of the following variables: 1) fever of more than 38 °C (100.4 °F) or less than 36 °C (96.8 °F), 2) heart rate of more than 90 beats per minute, 3) respiratory rate of more than 20 breaths per minute or arterial carbon dioxide tension (PaCO_2_) of less than 32 mmHg, 4) abnormal white blood cell count (> 12,000/μL or < 4000/μL or > 10% immature [band] forms) [24, 25]. Septic shock was defined as sepsis associated with organ dysfunction and persistent hypotension despite volume replacement [24, 25].

According to international guidelines [26–28], blood cultures were collected simultaneously from both central line and peripheral blood.

So doing, we identified five different mutually exclusive conditions: 1) significant different time to positivization of blood cultures (at least 2 h) between central line sample and peripheral sample (sepsis related to CVC – CLABSIs); 2) not significant different time to positivization of blood cultures (sepsis not related to CVC); 3) positive blood cultures from central line sample and negative from peripheral one (CVC colonization); 4) negative blood cultures from central line sample and positive from peripheral one (contaminated blood cultures); 5) negative blood cultures from both peripheral and central line samples (SIRS not sustained by sepsis). CVCs were removed when conditions 1 and 3 occurred. Catheter tips were collected and prepared for subsequent microbiological analysis and identified microbial species have been reported.

Each catheter was designated by type of vessel used (peripheral versus central); site of insertion (subclavian, femoral, internal jugular, peripheral, and Peripherally Inserted Central Catheter [PICC]) [10, 16].

Moreover, for each patient, data concerning provenance, parenteral nutrition, presence of tracheostomy tube and mechanical ventilation were collected.

Finally, in order to measure the severity of clinical condition of each patient, we adopted APACHE III score [29] and Charlson’s Comorbidity Index score [30].

### Microbiological methods

The entire medical center is served by a central microbiology laboratory, which is open from 7:00 AM to 7:00 PM, Monday through Saturday.

For adult patients with suspected BSIs, the center’s standard of care requires the sequential collection at 30-min intervals of at least three sets of aerobic and anaerobic BCs (CLSI). For each set, a 20-mL blood sample is collected via a single venipuncture or intravascular line access. Skin or access ports are disinfected with alcohol and povidone iodine.

The blood sample is used to inoculate one BACTEC Plus Aerobic/F and Anaerobic bottles (10 mL of blood each) (Becton Dickinson Instrument Systems, Sparks, Md). The bottles are brought to the laboratory and incubated up to 5 days in the BACTEC FX automated blood culture instrument (Cultures arriving when the laboratory is closed are stored at room temperature in accordance with manufacturers’ instructions). When the growth index of a bottle was positive, broth aliquots were collected for standard identification studies, which entailed Gram staining (the results of which were immediately reported to the patient’s physician), routine subculture, and matrix-assisted laser desorption ionization–time of flight (MALDI-TOF) mass spectrometry (MALDI BioTyper, Bruker Daltonik GmbH, Leipzig, Germany) analysis of culture samples, supplemented when necessary with additional biochemical methods and/or 16S rRNA gene sequencing [31, 32]. Antibiotic susceptibility tests were performed using the Vitek 2 system (bioMérieux, Marcy l’Etoile, France). Confirmatory MIC testing for oxyimino-cephalosporins and carbapenems was carried out by Etest (bioMérieux). Results were interpreted according to the European Committee on Antimicrobial Susceptibility Testing (EUCAST) breakpoints (EUCAST table). Catheter tip cultures were performed according to the method described by Maki et al. [33].

### Study design

The study was divided in two periods of equal duration time (9 months): a preliminary prospective observational period (phase I) and a subsequent prospective interventional period (phase II).

Patients enrolled during phase I were the Treat 0 group. During phase II, two interventional strategies have been adopted:

1. at the beginning of interventional study and then every 45 days, physicians and nurses of RICU have been trained and retrained to GAVECELT (“Long Term Venous Central Lines” Open Group – https://gavecelt.it/nuovo/) “bundle” recommendations concerning the management of CVC. The bundle includes: hand hygiene and precautions for protection and safety, adequate insertion site, echo-guided placement of central venous line, use of clorexidine 2% for skin disinfection of insertion site chosen and subsequent continuous or discontinuous disinfection of exit site, use of suture-less devices, use of transparent semi-permeable dressing whenever applicable and immediate removal of catheter when no longer needed. Moreover, in order to reduce the use of unnecessary catheters, we regularly evaluated the need for CVCs for both patients admitted to RICU and patients moved out of the ICU and unnecessary catheters were promptly removed.
2. After the first training meeting, the use of Curos® Disinfecting Port Protector for needleless valves port-protector (CUROS, 70% isopropyl alcohol-impregnated, Ivera Medical, San Diego, California, US) has been introduced.

Patients ruled in during the interventional period have been randomized into two groups:

1. patients with CVC managed by healthcare workers trained/retrained to GAVECELT recommendations (Treat 1 group).
2. Patients with CVC managed by healthcare workers trained/retrained to GAVECELT recommendations with the aid of port protector devices (Treat 2 group).

Curos® Disinfecting Port Protector is a passive disinfection device that luer-locks securely onto needleless IV ports to disinfect in 3 min. If not removed, ports stay clean and protected for 7 days. The Curos® is intended for use on swabbable luer access valves as a disinfecting cleaner prior to line access and to act as a physical barrier to contamination between line accesses. Curos disinfects the valve three minutes after application and acts as a physical barrier to contamination for up to seven days if not removed.

### Analysis

Description of the sample was made calculating mean values and standard deviation for continuous variables, percentages for dichotomous or ordinal variables.

Simple randomization was the method used (Stata version 9, StataCorp LLC, USA).

Regarding concealment, a centralized computer algorithm was adopted to assign subjects to Treat 1 or Treat 2 group.

The head of nurses (MC) and physicians allocated to the intervention groups (GP, DM, GM) were aware of the allocated arm, outcome assessors and data analysts (RI, GS, GMC, RT, TS) were kept blinded to the allocation.

Overall catheter dwell time was reported. CLABSIs rate expressed as number of sepsis related to CVC per 1000 days of catheter dwell time was computed. Data concerning number of CVC colonizations, contaminated blood cultures and negative blood cultures have been also reported.

The primary analysis was a quasi-experimental design comparing baseline rate of outcome measurements (the rates of CLABSIs, CVC colonizations and contaminated blood cultures) to the rates during the intervention. Then, the same measurements were compared between the two interventional groups (educational alone and combined with port protector).

Incidence of primary and secondary outcomes were analyzed using log rank test and Cox regression.

Variables included in the model were: type of treatment (educational interventions and educational interventions + Curos compared to the observational period), site of insertion, APACHE III score, Charlson Comorbidity Index, provenance of patient, hospital days before the admission to RICU, presence of tracheostomy, length of stay on mechanical ventilation, age and sex.

## Results

One hundred thirty-two patients were enrolled: 86 during the observational period (phase I) and 46 during interventional period (phase II), the latter divided randomly into two study groups: 25 in study group of educational interventions (Treat 1 group) and 21 in study group of educational interventions + Curos (Treat 2 group) (see Consort 2010 Flow Diagram).

Table 1 shows demographic characteristics of study population divided into three study groups. The three study groups did not differ in severity of clinical condition, assessed by APACHE III score and Charlson Comorbidity index, percentage of patients on mechanical ventilation, also through tracheostomy. Instead, there is a higher percentage of patients on parenteral nutrition in study group 0. The central venous accesss were divided in PICC, venous access placed in the subclavian vein / internal jugular vein and venous access placed in the femoral vein. Table 1 describes the distribution of type of venous catheter in the three study groups considered.

**Table 1:**
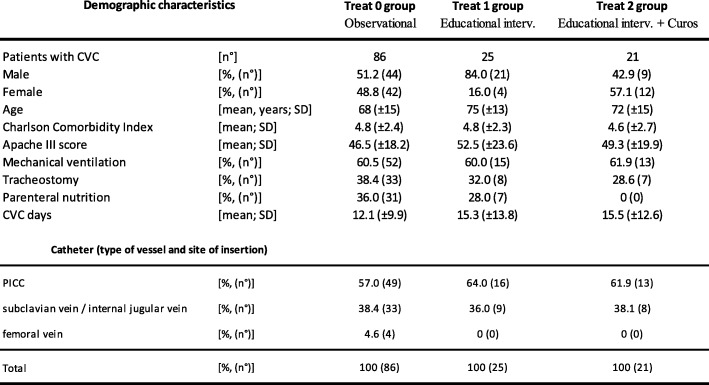
Demographic characteristics of the study population.

Table 2 describes rate of CLABSIs, colonizations of CVCs and contaminated blood cultures during observational period (phase I) and interventional period (phase II).

**Table 2:**
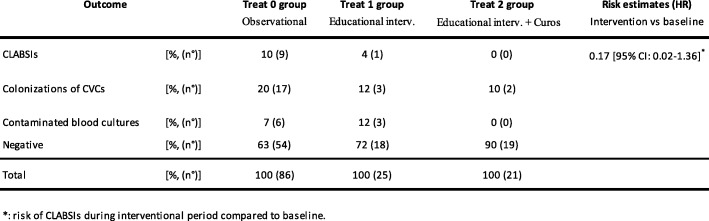
The rate of CLABSIs, CVC colonizations and contaminations of blood cultures.

During the first period, rate of CLABSIs (expressed as CLABSIs / 1000 catheter days of life) was 8.6.

The measures implemented during interventional period led to a reduction of rate of both CLABSIs and colonizations of venous catheters.

**Fig. 1:**
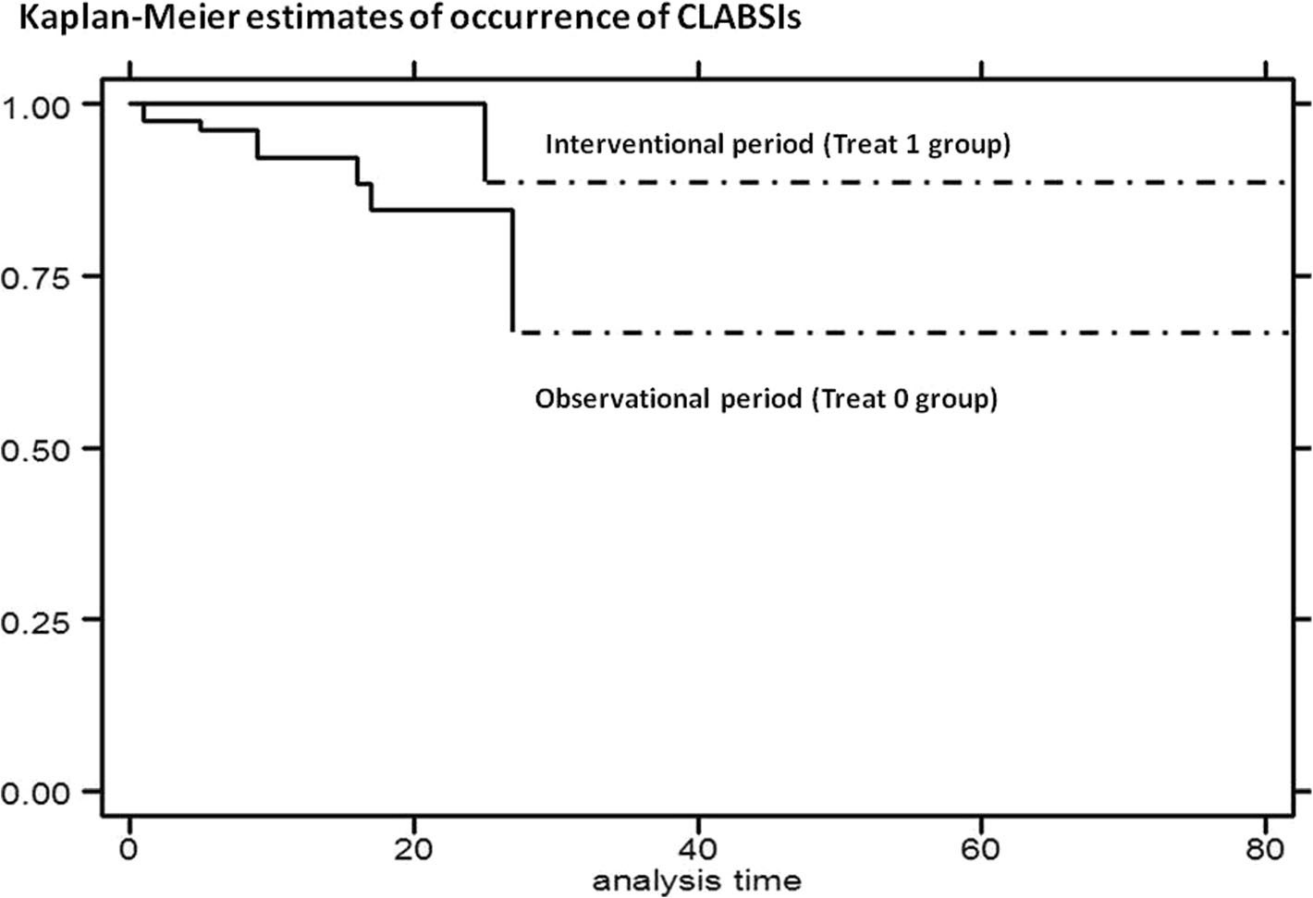
Kaplan-Meier curves: estimates of occurrence of CLABSIs by treatment: interventional period (Treat 1 group) compared with observational period (Treat 0 group)

In particular, educational measures combined to device led to zero the cases of CLABSIs. The comparison between two periods has detected a reduction of incidence of CLABSIs at the margin of significance [log rank test, *p* = 0.0568]. Moreover, risk of CLABSIs reduced during interventional period, with the hazard ratio (HR) of 0.17 [95% Conf. Interval: 0.02–1.36, *p* = 0.096] [Cox regression – Breslow method] [Fig. 1].

Subsequently, we focused on patients with colonization of venous catheter.

First, we performed univariate analysis (log-renk test) to evaluate incidence and, subsequently, risk of colonization according to treatment, excluding cases of CLABSI and comparing patients with CVC colonization with ones with contamination of blood cultures and negativity of microbiological investigations (negative).

**Fig. 2:**
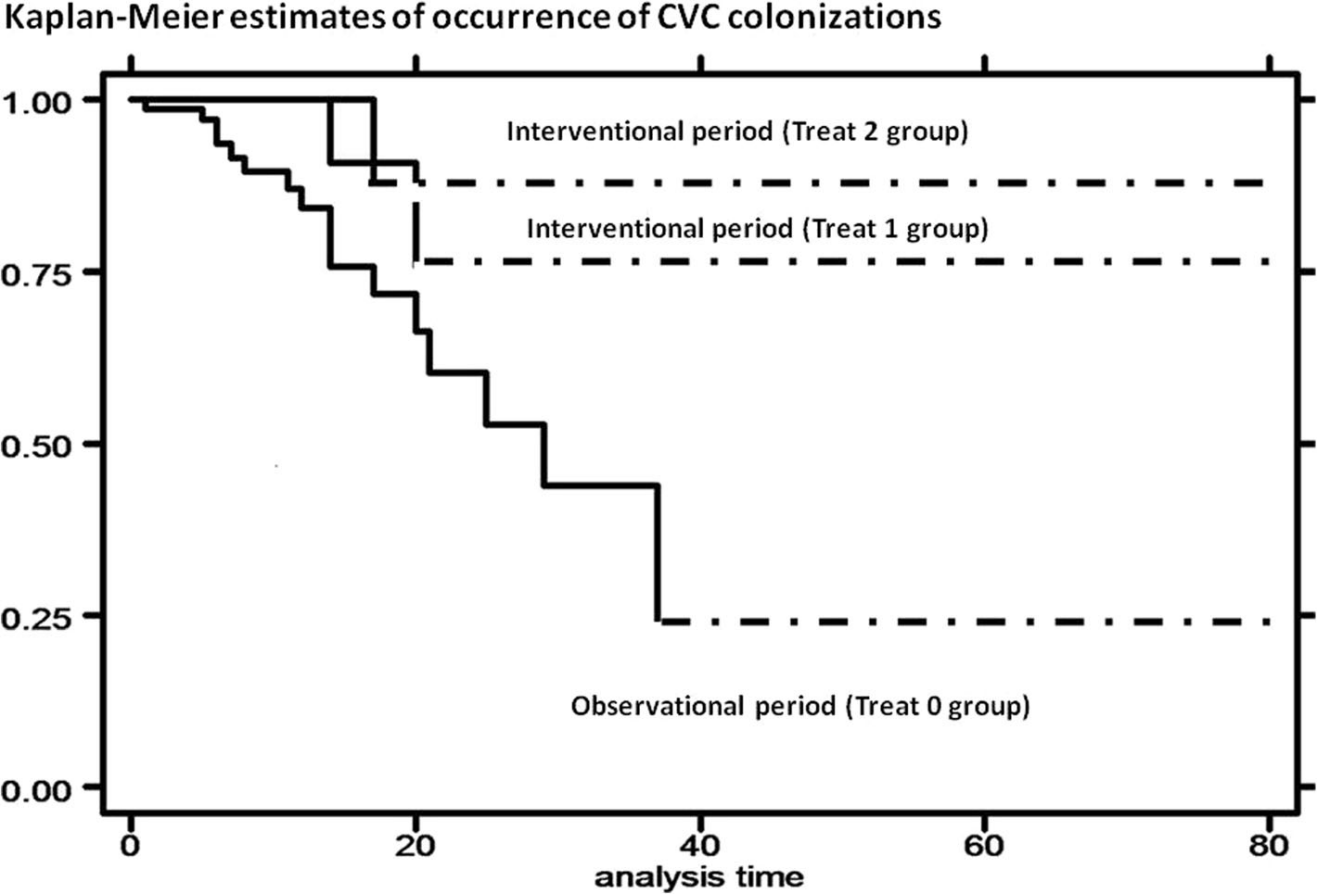
Kaplan-Meier curves: estimates of occurrence of CVC colonization by treatment: interventional period (Treat 1 group and Treat 2 group) compared with observational period (Treat 0 group)

During prospective interventional period, implemented treatments led to a reduction of incidence of colonization [log rank test, *p* = 0.0635]. Moreover, risk of colonization reduced in both treatment groups during interventional period, with HR of 0.30 [95% Conf. Interval: 0.07–1.29] for Treat 1 group (educational interventions) and HR of 0.33 [95% Conf. Interval: 0.07–1.45] for Treat 2 group (educational interventions + Curos) [Cox regression – Breslow method] [Fig. 2].

Subsequently, we evaluated the distribution of cases of CVC colonization according to site of insertion and the risk of colonization of CVC with Cox regression – Breslow method analysis [Table 3].

**Table 3:**
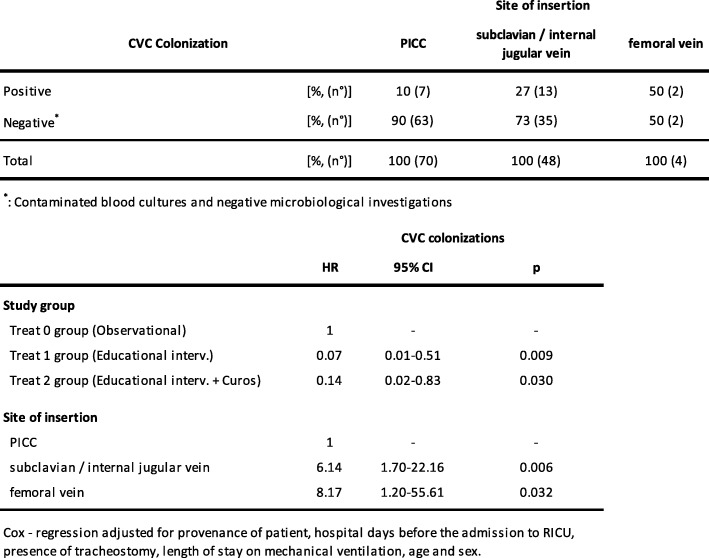
CVC colonizations according to the site of insertion.

It is possible to observe that risk of colonization of CVC significantly reduced in both interventional groups (HR: 0.07, 95% Conf. Interval: 0.01–0.51 for Treat 1 group – educational interventions; HR: 0.14, 95% Conf. Interval: 0.02–0.83 for Treat 2 group – educational interventions + Curos). Conversely, there was a significantly increased risk of colonization of CVC for patients to whom venous catheter was placed in subclavian / internal jugular vein and femoral vein with HR of 6.14 and 8.17, respectively.

**Fig. 3:**
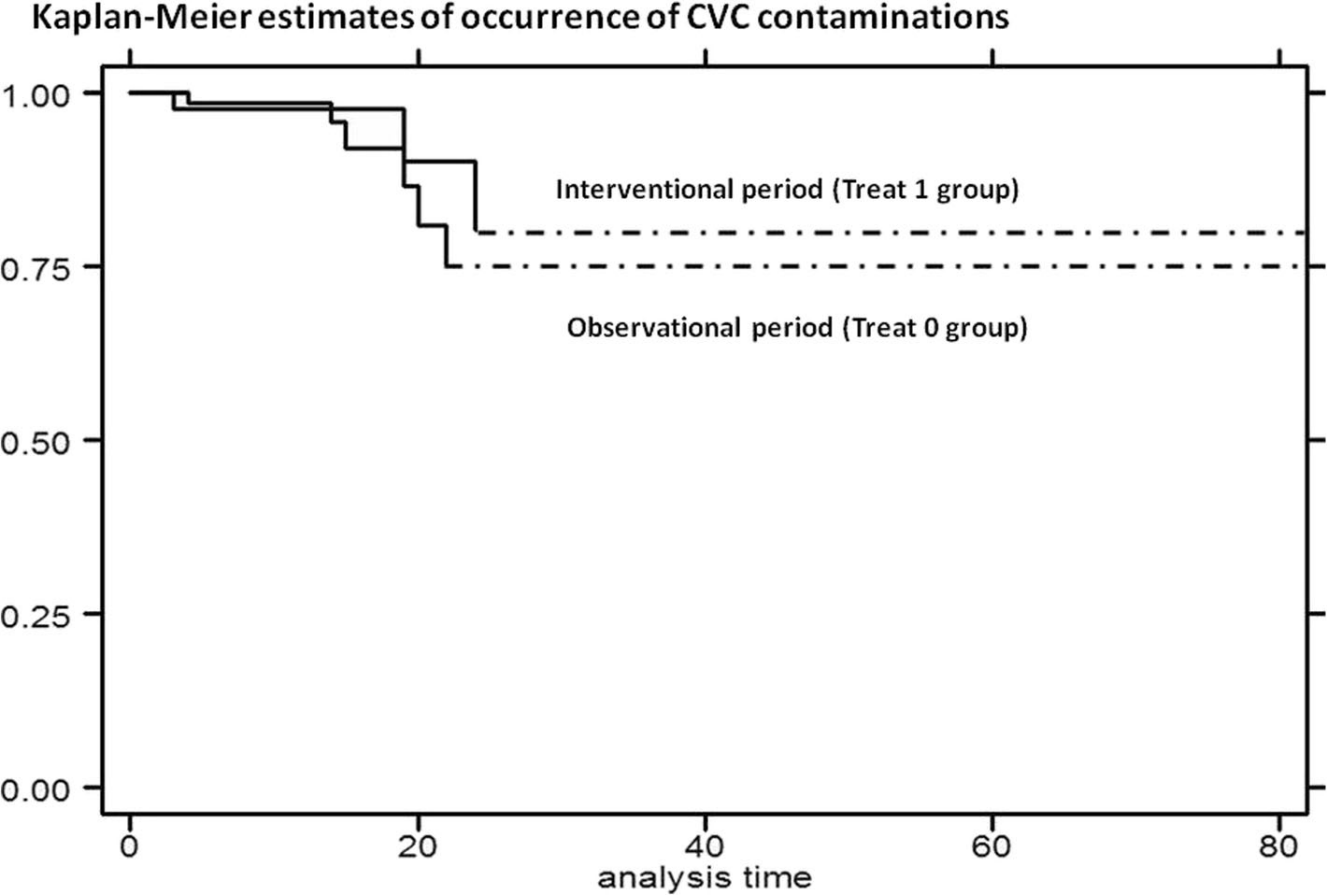
Kaplan-Meier curves: estimates of occurrence of CVC contamination by treatment: interventional period (Treat 1 group) compared with observational period (Treat 0 group)

Finally, we observed that use of educational measures combined to application of device led to zero cases of contaminated blood cultures [Table 2]. Nonetheless, there was no significant reduction between two study periods [log rank test, *p* = 0.6139] [Fig. 3].

The organisms recovered are presented in Table 4.

**Table 4:**
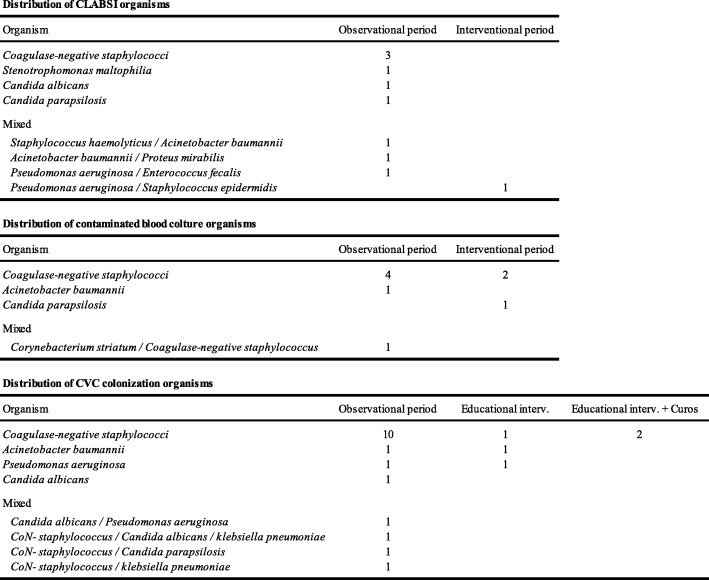
Distribution of CLABSI organisms, CVC colonization organisms and contaminated blood culture organisms.

## Discussion

In this prospective randomized study, we explored rate of CLABSIs, CVC colonizations and contaminated blood cultures before and after introduction of educational interventions alone and combined with port protector as adjuvant tool.

To the best of our knowledge, this is the first study performed in a RICU.

Three main conclusions can be extracted from this work. (i) educational interventions combined to Curos® Disinfecting Port Protector led to zero rate of CLABSIs. Additionally, risk of CLABSIs reduced during interventional period. (ii) The measures implemented reduced also rate of colonizations of the venous catheters. Lastly, (iii) risk of colonization of CVC increased for patients to whom venous catheter was placed in subclavian / internal jugular vein and femoral vein.

The importance of the education of healthcare personnel regarding the indications for intravascular catheter use, proper procedures for the insertion and maintenance of intravascular catheters, and appropriate infection control measures to prevent intravascular catheter-related infections is well known [34]. Furthermore, previous studies also highlighted the need of periodical assessment of knowledge of and adherence to guidelines for all personnel involved in insertion and maintenance of intravascular catheters [34, 35].

Recently, Sweet MA et al. [18] showed that alcohol-impregnated port protectors and needleless neutral pressure connectors significantly reduced rates of CLABSIs and contaminated blood cultures in an oncology patient population. Similar results were reported by Wright MO et al., [36] where disinfection cap with 70% alcohol reduced line contamination, organism density, and CLABSIs.

The novelty of our study consists in performing a prospective study with the specific aim of assessing effects of educational interventions combined to alcohol-impregnated port protector on rate and risk of CLABSIs, CVC colonizations and contaminated blood cultures in a clinical setting at high risk of bloodstream infections.

This strategy allowed us to zero rate of CLABSIs (from 8.6 CLABSIs / 1000 catheter days of life to 2.6 with educational interventions and zero with educational interventions combined to port protector). Our experience demonstrates that this goal, warmely supported by international experts [10], is feasible.

Another interesting observation is that this preventive strategy reduced also risk of CLABSIs (83% reduction).

Strong evidence exists in literature about reduction of risk for infection following standardization of aseptic care [34, 35]. Moreover, specialized “IV teams” reduced incidence of catheter-related bloodstream infections, associated complications, and costs [37].

In our hospital, there is a specialized IV team dedicated to the CVC placement. Therefore, we preferred to focus our attention on management of catheter essentialy based on training and retraining of physicians and nurses of the RICU to GAVECELT “bundle” recommendations.

The reduction of both rate and risk of CLABSIs confirms previous reports [9, 38] asserting the catheter hub colonization as the primary pathogenetic explanation of central catheter infections after the insertion (intraluminal causal pathway).

In our study, both treatments caused a decrease of rate of CVC colonizations (70% reduction with educational interventions and 67% reduction with educational interventions combined to port protector).

This is in line with the observations by Wright MO, who found that the catheter hub contamination was significantly less likely to occur, and, when it did occur, the number of recovered organisms were significantly fewer with the use of the disinfection cap [36].

Subsequently, we observed that risk of colonization of CVC significantly reduced in both interventional groups (93% reduction with educational interventions and 86% reduction with educational interventions and Curos). Conversely, there was a significantly increased risk of colonization of CVC for patients to whom venous catheter was placed in subclavian / internal jugular vein (HR of 6.14) and femoral vein (HR of 8.17) compared to patients to whom a PICC was placed.

Our study confirms that site at which a catheter is placed influences the subsequent risk for catheter-related infection. The influence of site on the risk for catheter infections is related in part to the density of local skin flora.

As far as we know, this is the first study that prospectively compared infection rates for catheters placed in jugular, subclavian, femoral and peripheral veins (PICC). In agreement with literature [39], our results highlight that femoral catheters have highest colonization rates.

Finally, we observed that educational measures combined to port protector led to zero the cases of contaminated blood cultures.

This study has some limitations. First, sample size is apparently limited if compared to study period (18 months). Our RICU works essentially as “step down” unit for difficult to wean patients coming from our ICUs (more than 50% of enrolled patients). Moreover, our unit provides multidisciplinary rehabilitation and serves as a bridge to home-care programs or long-term care facilities. Mean length of stay in our unit was 33 days and this partially explains the number of enrolled patients.

Furthermore, number of patients enrolled during interventional period was lower than that of the observational period. We believe that this apparent limitation reflects a better selection of patients who will benefit from the use of catheters due to educational training/retraining, thus this finding can be considered a positive result of the study.

## Conclusions

This study shows that synergistic effects of educational interventions and use of Curos® Disinfecting Port Protector allow to zero rate and reduce the risk of CLABSIs in a Respiratory semi-Intensive Care Unit.

## Abbreviations

CLABSIs: Central Line-Associated BloodStream Infections
CRBSIs: Catheter-related BSIs
CVC: Venous central catheter
EUCAST: European Committee on Antimicrobial Susceptibility Testing
HR: Hazard ratio
ICU: Intensive Care Unit
PICC: peripherally inserted central catheter
RICU: Respiratory semi-Intensive Care Unit
SIRS: Systemic inflammatory response syndrome
